# Humidity-Controlled Tunable Emission in a Dye-Incorporated Metal–Hydrogel–Metal Cavity

**DOI:** 10.1021/acsphotonics.2c00202

**Published:** 2022-06-23

**Authors:** Dipa Ghindani, Ibrahim Issah, Semyon Chervinskii, Markus Lahikainen, Kim Kuntze, Arri Priimagi, Humeyra Caglayan

**Affiliations:** Tampere University, Faculty of Engineering and Natural Sciences, 33720 Tampere, Finland

**Keywords:** metal−insulator−metal system, hydrogels, fluorescent dye, humidity, tunable emission, stimuli-responsive materials

## Abstract

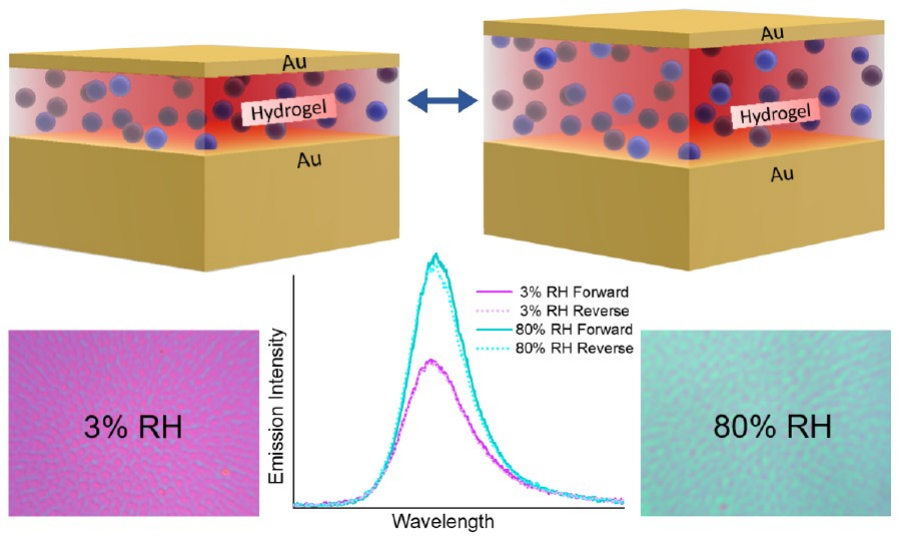

Actively controllable photoluminescence is potent for a wide variety of applications from biosensing and imaging to optoelectronic components. Traditionally, methods to achieve active emission control are limited due to complex fabrication processes or irreversible tuning. Here, we demonstrate active emission tuning, achieved by changing the ambient humidity in a fluorescent dye-containing hydrogel integrated into a metal–insulator–metal (MIM) system. Altering the overlapping region of the MIM cavity resonance and the absorption and emission spectra of the dye used is the underlying principle to achieving tunability of the emission. We first verify this by passive tuning of cavity resonance and further experimentally demonstrate active tuning in both air and aqueous environments. The proposed approach is reversible, easy to integrate, and spectrally scalable, thus providing opportunities for developing tunable photonic devices.

## Introduction

Manipulating the optical properties of an emitter is of paramount importance for developing efficient light sources for advanced nanophotonic devices,^1,2^ fluorescence microscopy,^3^ and various optoelectronic applications.^4^ Over the past decades, numerous efforts have been made to control the emission properties of organic dye molecules, owing to their high photoluminescence (PL) quantum yield and broadband emission,^5^ paving the way for new solutions for photonic devices such as LEDs,^6^ lasers,^7^ and single-photon sources.^8^ To improve the efficiency and functionalities of the photonic devices, it is highly desirable to control and enhance the emission properties of the photonic structures. In addition, a facile, real-time, reversible, and actively tunable luminescence system is of great importance, as it serves as the basis for next-generation photonic elements. Furthermore, on-demand control of the emission will enable broadband sensing and full-color display devices. To date, plasmonic^9^ and dielectric cavities^10,11^ are routinely utilized to boost the PL signals by increasing the local density of photonic states^12,13^ around the emitter. For instance, thin metallic films and plasmonic nanoantennae tightly confine the electromagnetic field into a small volume due to plasmonic resonance. Similarly, high-index dielectric nanostructures, such as 1D gratings,^14^ 2D photonic crystals,^15,16^ and dielectric resonators,^17,18^ localize the electromagnetic fields by Mie-resonances. Although these approaches have shown notable PL enhancement, they bring along some limitations. First, the resonance modes corresponding to these resonators are subwavelength, which require careful alignment of dye molecules with the resonance hot spots. Secondly, continuous tuning of resonance frequency in plasmonic and dielectric resonator systems possesses fabrication complexity. In addition to the PL enhancement, its active tunability is also important. Several ways exist to actively tune the emission properties. These include the application of magnetic,^19,20^ electric,^21^ and optical^22,23^ fields. Furthermore, a diverse range of tunable metasurfaces based on mechanical actuation,^24−26^ phase-change materials,^27−31^ and liquid crystals^32−34^ exist, offering dynamic tuning of optical properties. For a metasurface, the collective response of subwavelength-sized resonators is primarily determined by the resonator geometry and size.^35,36^ Integrating the emitter into a tunable metasurfaces would allow for active emission tuning. However, all these approaches either require complex experimental setup, fabrication techniques, lack reversibility, or exhibit small spectral tunability.^37^ In addition, complex and time-consuming fabrication procedures hamper their translation into real-world applications. To circumvent this, we utilize a metal–insulator–metal platform for tuning the emission.

The metal–insulator–metal (MIM) structure is relatively simple and exhibits high-quality-factor resonance.^38^ The resonance wavelength of the MIM cavity is scalable by simply changing the thickness of the insulating layer. We exploited this property to demonstrate an active emission tuning using a hydrogel as the insulating layer. Hydrogel is a stimuli-responsive hydrophilic cross-linked polymer network capable of holding a large amount of water in its network. The volume and the optical and mechanical properties of the hydrogels can be changed due to the reversible swelling–deswelling process in a humid and aqueous environment.^39^ An increment in humidity allows the hydrogel to absorb water from the environment and swell, resulting in a relatively large thickness change of the thin hydrogel.^40,41^ Due to this remarkable feature, hydrogels have stood out as promising materials for developing actively tunable plasmonic devices.^42−44^

To showcase the active emission tuning, we integrated a photoluminescent hydrogel, obtained via covalent functionalization with rhodamine B (RhB) as an emitter (see the Methods section for further details) into a MIM cavity as a tunable platform. Our results reveal an active emission tuning by varying the thickness of the hydrogel, resulting in maximum emission enhancement when the MIM cavity resonance overlaps with both the absorption and emission bands of the emitter. We demonstrate humidity-responsive PL of the emitter coupled to an actively tunable MIM cavity at room temperature, via both passive and active tuning schemes. In the passive tuning, we fabricated MIM cavities with different thicknesses of the hydrogel-based insulating layer, while for the active tuning, the hydrogel thickness was varied in response to humidity or the presence of water. We envision that our study will pave the way for engineering light–matter interactions, advancing the fundamental understanding and in the longer-term potentially contributing to the technological development of luminescent devices. The proposed solution exhibits real-time tunability, reversibility, and large spectral tuning of the cavity resonance with relatively easy fabrication and experimental setup. The planar topography and scalability of our structure will enable large-area devices to function at the desired spectral region, being well-positioned to enable tunable light sources.

## Results and Discussion

### MIM (Metal–Hydrogel+Dye–Metal) Design

**Figure 1:**
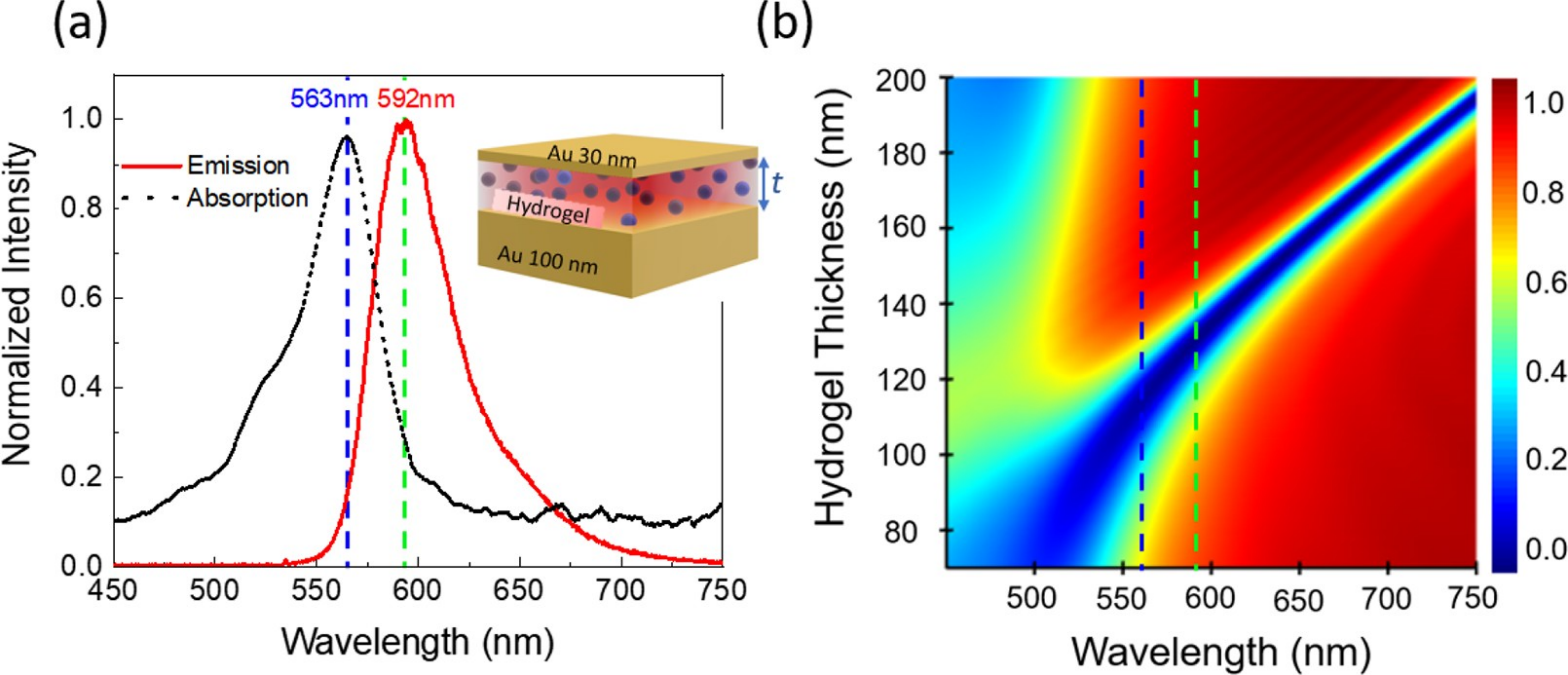
(a) The absorption and emission spectra of RhB are presented as black dotted and red solid curves, respectively. Inset: schematic representation of the RhB-containing hydrogel incorporated into the MIM device. (b) Simulated reflection from the MIM for different hydrogel thicknesses.

The simultaneous overlap of dye’s emission and absorption bands with the cavity resonance leads to enhanced emission. This arises from a combination of the Purcell effect and the excitation rate enhancement.^45^ This guideline defines the design of the particular MIM structure, especially the dielectric thickness to enhance the emission. The measured absorption and emission of RhB depicted in Figure 1a show a small Stokes shift of ≈30 nm. The black dotted curve represents the absorption spectrum, peaking at 563 nm, which is marked with a blue dashed line, and the red solid line is the emission of RhB with a peak at 592 nm, highlighted using the green dashed line. As Figure 1a suggests, the wavelength range of 540 to 650 nm is optimal for simultaneous overlap of the cavity resonance with both the emission and absorption of RhB. To identify the suitable thickness range of the dielectric layer of the MIM structure, we utilized both the dye characteristics and the MIM cavity resonance presented in Figure 1. The latter is obtained between the required wavelength region when the dielectric thickness is between ≈90 and ≈150 nm.

The MIM design and the thickness of the hydrogel layer were obtained using numerical simulation software based on the finite-difference time-domain (FDTD) method. The hydrogel was modeled with a refractive index *n* = 1.503,^46^ and further simulation details are given in Methods. The simulated reflection for the MIM cavity at varying hydrogel thicknesses is shown in Figure 1b, and the positions of absorption and emission maxima of RhB are shown by blue and green dashed lines, respectively.

In order to realize emission-tunable MIM, we deposited a gold (Au) layer using an e-beam evaporator and confirmed its thickness with a stylus profilometer under cleanroom conditions. The insulator layer, the poly(*N*-isopropylacrylamide)-acrylamidobenzophenone (PNIPAm-BP) hydrogel with pendant RhB molecules, was spin-coated on the bottom Au layer and cross-linked with a UV light (see Methods for more details). The thickness of the hydrogel was varied to tune the cavity resonance using different spin coating conditions. The schematic of the MIM cavity is shown in the inset of Figure 1a, where the thicknesses of the top and bottom metallic layers are 30 and 100 nm, respectively. The bottom layer serves as a reflector while the top layer is partially transparent, allowing to collect the reflected light. Au has been used for the metal layers owing to its nonoxidizing nature and stable plasmonic properties.^47^ We have selected PNIPAm-BP as the insulating layer owing to its excellent thin-film forming^41,48^ and swelling properties.^49,50^ Because of the thin hydrogel layer, the swelling/deswelling modulates the hydrogel thickness, hence changing the resonance of the whole MIM structure.^51^

### Passive Emission Tuning

**Figure 2:**
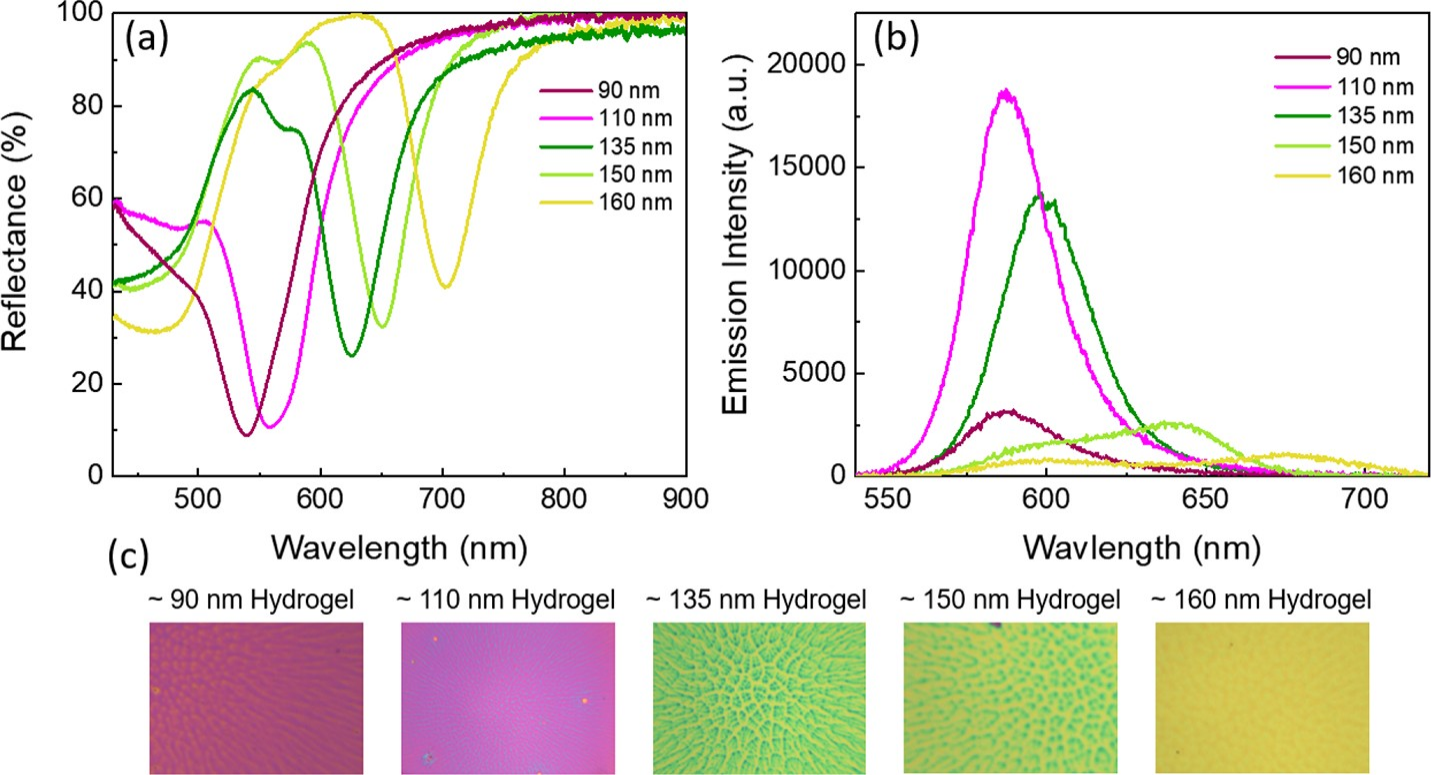
(a) Measured reflectance spectra for the MIM cavities with different hydrogel thicknesses. (b) Steady-state PL spectra of RhB in the different MIM cavities. (c) Optical images of the MIM samples with different hydrogel thicknesses exhibit different bright colors as per their reflectance. The thicknesses of hydrogels were measured with profilometer.

To demonstrate passive (i.e., without real-time control) emission tuning by varying the thickness of the MIM, we investigated the effect that the overlap between the cavity resonance and the absorption and emission of the RhB dye has on the emission intensity, by using MIM cavities with dry hydrogel thicknesses from ≈90 to ≈160 nm. Here, the dry hydrogel thickness implies its thickness at a relative humidity of 28%. We measured the reflectance and emission from the samples using a 20× air objective as detailed in the Methods section. Figure 2a shows the measured reflectance spectra for the MIM cavities with various hydrogel thicknesses. With an increase in the hydrogel thickness, the MIM cavity resonance redshifts, which is also evident from the change in the color of the fabricated samples, as shown in Figure 2c.

Figure 2b shows the tunable emission of the emitters coupled with different resonance cavities of the MIM structures for passively varying the dry hydrogel thickness to tune the spectral overlapping. The MIM cavity with a dry hydrogel thickness of 90 nm exhibits cavity resonance at 540 nm, which strongly overlaps with the absorption spectrum of RhB and barely with its emission spectrum. This yields very low emission intensity, as shown by the magenta solid line in Figure 2b. Upon increasing the hydrogel thickness to 110 nm, the cavity resonance redshifts and lies at 558 nm (Figure 2a, pink solid line), exhibiting a significant overlap with both the absorption and the emission spectra of RhB. This leads to an opportunity to exploit both the Purcell factor enhancement and the excitation rate enhancement to boost the overall emission of the system.^45^ The simultaneous contribution from both processes yields the maximum PL intensity, and we observed the highest emission as shown by the pink solid line in Figure 2b (enhancement factor of ≈7 as compared to the dry thickness of 90 nm). For the sample with a dry hydrogel thickness of 135 nm, where the cavity resonance overlaps with the emission and only slightly with the tail of the absorption, the emission further redshifts, and its intensity decreases. We also further increased the dry hydrogel thicknesses to 150 nm, which shifted the cavity resonance away from the absorption and emission of RhB, with only a minor overlap with the emission tail. As a result, we observed further reduction of emission intensity with a small shoulder at about 650 nm, as shown in Figure 2b.

We would like to highlight that the underlying principle behind the emission intensity change is either the Purcell factor enhancement (when the cavity resonance overlaps with only the emission band) or both the Purcell enhancement and the excitation rate enhancement (when the cavity resonance overlaps with the absorption and emission bands simultaneously). Since the Stokes shift for RhB is small, it is difficult to completely isolate the contributions arising from the Purcell factor and the excitation rate enhancement. Therefore, the wavelength corresponding to the maximal emission enhancement slightly offsets the cavity resonance wavelength.

### Reversible and Active Emission Tuning in Air

Serpe et al. have worked extensively on tunable etalons where the dielectric layer comprises a PNIPAm microgel, which in an aqueous environment responds to different stimuli and displays color tuning.^52−58^ In addition, Chervinskii et al. have shown the dynamic tuning of the MIM cavity resonance by tuning the thickness of a hydrogel insulating layer.^48^ However, all aforementioned studies do not converse emission tuning. Herein, after first exploring the passive tuning of emission, we demonstrate active tuning of emission via hydrogel-based MIM in response to ambient humidity. The humidity-based tuning allows controlling the overlapping region with the emission and absorption of RhB, which manifests as tunable emission.

**Figure 3:**
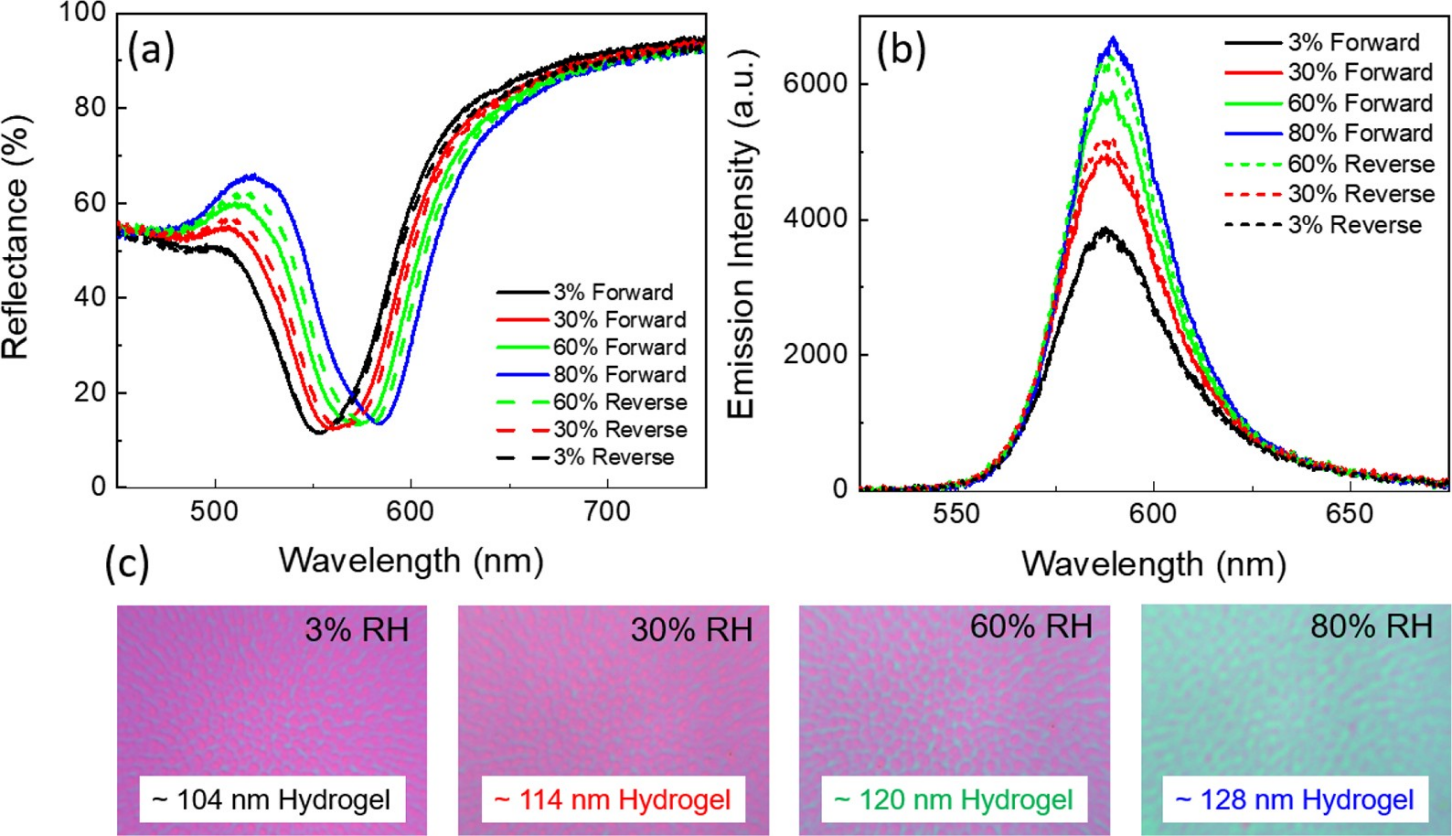
(a) Measured reflectance spectra of MIM with dry hydrogel thickness 110 nm at different humidities. Solid curves show the forward cycle (humidity increases from 3% to 80%) and dashed curves represent the reverse cycle (humidity reduces from 80% to 3%). (b) Steady-state PL spectra of the RhB embedded in the MIM cavity at different humidities. (c) Optical images of the MIM sample at different humidities. The labeled thicknesses are extracted from the simulation.

The passive tuning study revealed 110 nm (see Figure 2b) as the optimized hydrogel thickness for enhancing the emission intensity. Therefore, for demonstrating the active tuning, we selected the MIM with a dry hydrogel thickness of 110 nm. We used a humidity-controlled chamber to measure the PL spectra of the structures, using a customized experimental setup with controlled relative humidity (RH) and performed reflectance and PL measurements (see Figure 3a,b) of the samples in the controlled environment. The measurements were conducted at 3%, 30%, 60%, and 80% RH. Furthermore, measurements were taken in a reversible manner (i.e., from 80% back to 3% RH value) to attain the reversibility of the PL response. Figure 3c shows the optical micrographs of the MIM sample at different RH values. We observed distinct color changes in the samples with the increment of the RH value. This signifies that with the increase in humidity, the hydrogel thickness changes, which in turn modifies the cavity resonance wavelength. We note that the swelling of the hydrogel is strongly dependent on its initial thickness and uniformity. Due to defects present in the spin-coated samples, the swelling process is inevitably somewhat nonuniform. To minimize the nonuniformity, an alternative solution of two-step spin coating was utilized.^41^

To quantify the swelling and the corresponding overlap with the RhB absorption and emission spectra, we extracted the hydrogel thickness from the simulation results. At a constant temperature, with an increase in RH from 3 to 80%, the hydrogel thickness changes from 104 to 128 nm. As a result, the cavity resonance red-shifted by 30 nm, from 548 to 588 nm, as shown in Figure 3a. During this process, the cavity resonance overlaps better with both the absorption and emission bands of the RhB. Hence, we observed an almost 2-fold increase in the emission intensity while increasing the humidity from 3% to 80%, as shown in Figure 3b. The increase in humidity does not directly affect the intrinsic properties of the covalently bonded dye in the hydrogel matrix. Therefore, this increase in emission is solely due to the significant overlap of the cavity with the dye’s absorption and emission.

The humidity-induced emission tuning is reversible, and by decreasing the RH from 80% to 3%, the original spectrum is retained. However, the reverse cycle (3.5 h) is slightly slower than the forward cycle (3 h), because the rate at which the hydrogel expels water and the rate at which it absorbs water are different.^59^ We observed that in the reverse cycle, the humidified sample (80% RH) started expelling the absorbed water gradually with a decrease in RH value, and the cavity resonance retains its original position, as shown in Figure 3a (black, red, and green dashed line). The decrease in the hydrogel thickness during the reverse cycle also allowed to retrieve the PL intensity, as shown in Figure 3b (black, red, and green dashed line). The swelling and deswelling of the hydrogel allowed the reversible humidity-induced tuning of the optical response of the MIM cavity and, hence, reversible control of the PL intensity.

### Active Emission Tuning in Water

The previous active tuning scheme was performed in the air where the change in humidity was controlling the hydrogel thickness. However, controlling the thickness of the hydrogel with humidity does not allow large thickness variations. Therefore, to find the limits of our system, we immersed the hydrogel-integrated MIM sample in water. By immersing the sample in deionized water for different time periods, we achieved various hydrogel thicknesses that were generally larger than in the air humidity-controlled case, which allowed us to tune the MIM cavity resonance with large spectral shifts compared to the humidity-based control.

**Figure 4:**
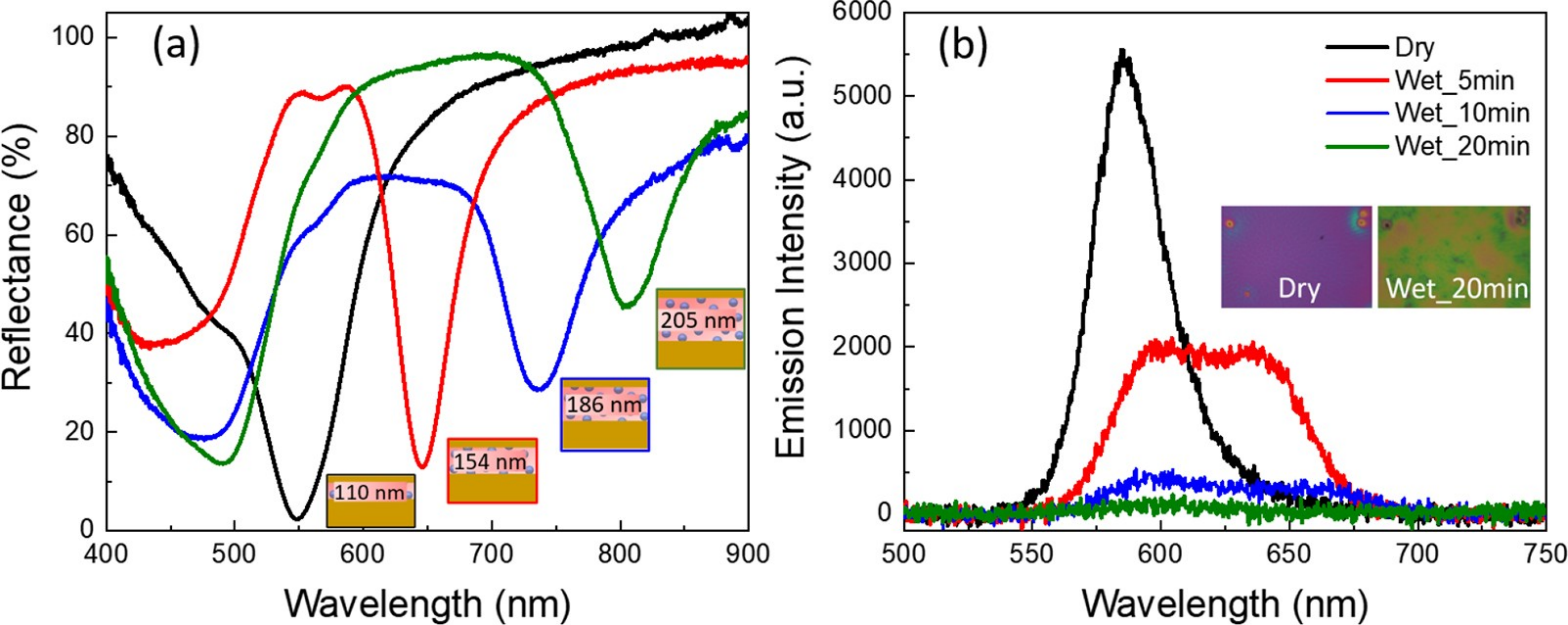
(a) Measured reflectance spectra of MIM with dry hydrogel thickness 110 nm when immersed in deionized water for 5, 10, and 20 min. Insets: the corresponding hydrogel thicknesses for each case, extracted from the simulation. (b) Steady-state PL spectra of MIM in dry case and when immersed in water for 5, 10, and 20 min. Inset: optical images of the sample in two cases: dry and after 20 min immersion.

We submerged the sample for 5, 10, and 20 min and monitored the change in the cavity resonance as well as the changes in the PL response of the dyes incorporated within the MIM cavity. Figure 4a shows the measured reflectance spectra of the MIM cavity with a dry hydrogel thickness of 110 nm and three submerged cases.

The inset of Figure 4b shows the optical images of the sample in the dry case, corresponding to a thinner hydrogel layer, and the wet case, where the hydrogel swells^51^ and shifts the MIM resonance to a higher wavelength. To evaluate the tuning range of MIM structures, we compared the dry case with the wet case. The dry hydrogel (ambient conditions) thickness was 110 nm. After immersing the sample in deionized water for 20 min, we identified a spectral shift of 255 nm from 550 to 805 nm, as shown in Figure 4a, with black and green solid lines, respectively. As the cavity resonance shifts to a higher wavelength, its overlap with the dye’s absorption and emission spectra decrease. As a result, we observed a decrease in the emission intensity, as shown in Figure 4b. Subsequently, for the swollen hydrogel thickness of 205 nm, in which the cavity resonance does not overlap with the dye’s emission at all, we observed negligible emission intensity as shown by the green solid line Figure 4b.

## Conclusion

We report active emission tuning based on emitters embedded within the PNIPAm hydrogel-based metal–insulator–metal device. We demonstrate a 30 nm spectral shift and significant tunability in PL intensity in response to the humidity stimulus. Our structures showed reversible behavior and almost reproduced the initial results by utilizing the deswelling property of hydrogels. The maximum resonance shift (255 nm) and emission tunability were obtained by immersion of incorporated PNIPAm hydrogel-based MIM structure in deionized water. This large spectral shift is remarkable and of great importance, especially for sensing applications. Our approach mitigates the complex fabrication challenges and is versatile in nature that potentially can be translated to a broad spectral range to achieve on-demand tunability by judiciously choosing various hydrogel thicknesses integrated with different dye molecules. We envision a wide range of opportunities in targets that require on-demand optoelectronic tunability, ranging from integrated circuits to flat optics. Our findings may also provide new possibilities in actively tunable reversible photonic devices and contactless optical sensors.

## Methods

### PNIPAm-BP-RhB Copolymer Synthesis

*N*-Isopropylacrylamide (NIPAm) and azobis(isobutyronitrile) (AIBN) were commercially available. NIPAm was used as received, and AIBN was recrystallized from methanol before use. Rhodamine B acrylate was synthesized from commercial rhodamine B as described below. Benzophenone acrylamide (BP) and the copolymer were prepared in a similar fashion to the procedures in our earlier publication,^48^ yielding the copolymer with composition 98.5:2.0:0.5 (NIPAm/BP/RhB), determined with ^1^H NMR (500 MHz, CDCl_3_).

Rhodamine B (500 mg, 1.04 mmol) and a droplet of *N*,*N*-dimethylformamide were stirred in dry dichloromethane (6 mL) at 0 °C under argon, and oxalyl chloride (128 μL, 1.5 mmol) was added dropwise. The mixture was stirred at 0 °C for 30 min, until gas evolution was not observed. This solution was added into a solution of 2-hydroxyethyl acrylate (115 μL, 1.1 mmol), dry triethylamine (415 μL, 3.0 mmol), and a catalytic amount of *N*,*N*-dimethyl-4-aminopyridine in dry dichloromethane (10 mL) and stirred under argon for 24 h. The crude mixture was purified by column chromatography (from pure ethyl acetate to 10% methanol/ethyl acetate) to yield the product (352.5 mg, 59%) as a violet-red crystalline solid. ^1^H NMR (500 MHz, DMSO-*d*_6_) δ 8.26 (d, *J* = 7.4 Hz, 1H), 7.92 (t, *J* = 7.4 Hz, 1H), 7.86 (t, *J* = 7.4 Hz, 1H), 7.51 (d, *J* = 7.4 Hz, 1H), 7.07 (dd, *J* = 9.5, 2.0 Hz, 2H), 6.99 (d, *J* = 9.2 Hz, 2H), 6.95 (s, 2H), 6.21 (d, *J* = 16.6 Hz, 1H), 5.87–6.02 (2H), 4.14–4.28 (2H), 3.87–4.01 (2H), 3.55–3.74 (8H), 1.20 (t, *J* = 6.9 Hz, 12H).

### Solution Preparation, Film Thickness Optimization, and Photopolymerization

PNIPAm-BP-RhB copolymer (this is the composition of the copolymer after polymerization) was dissolved in filtered (filtered using 0.2 um pore sized PTFE Teflon filter) 94% ethanol with concentrations of 20 and 30 mg/mL. Using the 20 mg/mL solution, we acquired 90 and 110 nm thicknesses. For higher thicknesses (135–160 nm), we utilized the 30 mg/mL solution. Different thicknesses were achieved by using different spin coating parameters and two solutions. For better dissolution of the copolymer in ethanol, the solutions were sonicated for 10 min. Then magnetic stirring was used at 1400 rpm, 50 °C for 1 h. The solutions were filtered through PTFE membranes with 0.45 μm pores, then spin-coated the solution on a glass sample to optimize the desired thickness of the hydrogel layer. We used dynamic two-step spin-coating: (1) 10 s at 150 rpm, 100 acceleration during which the PNIPAm-BP-RhB solution was dispensed and predistributed onto the sample; (2) 30 s at 2000 rpm/3000 rpm/4500 rpm, 1000 acceleration to form the final coatings. The deposition was followed by drying for 45 min at 50 °C in a vacuum. The next step was photopolymerization under UV light (365 nm from CoolLED pE-4000 focused on sample area), the time required for complete cross-linking of PNIPAm-BP-RhB copolymer was 40 min. This time was confirmed by the disappearance of the 301 nm peak in the optical transmittance spectra of the reference hydrogel coatings on glass.^48^

### FDTD Simulations

MIM design and the thickness of the insulator layer (hydrogel) were optimized using numerical simulation (Ansys Lumerical FDTD Solutions) based on the finite-difference time-domain (FDTD) method. In the simulation, the symmetric and antisymmetric boundary conditions were applied in the *x* and *y* directions to minimize the simulation time, while PML (perfectly matched layer) was used along the *z*-axis to remove the unwanted reflections. A plane wave was launched from the *z*-axis to excite the resonance cavity mode. The complex refractive index of Au layers was assigned from the “Johnson and Christy”^60^ data set that was in-build in the software material library. The hydrogel was modeled with a refractive index *n* = 1.503,^46^ which corresponds to the state where there is no water absorbed into the hydrogel. An increase in hydrogel thickness depicts the absorption of water in the hydrogel, and the refractive index of water will influence the effective index of the hydrogel–water complex. However, the change in effective refractive index is relatively small, and its effect on the resonance shift is quite minimal.

### MIM Sample Fabrication

The samples were fabricated on 1 cm × 1 cm fused silica substrates. First, the samples were cleaned by sonicating them in acetone, isopropanol, and deionized water for 10 min each. The fused silica substrates were blow-dried and treated with oxygen plasma for pristine clean substrates. Subsequently, the adhesive layer of 1 nm Ti, followed by a 100 nm layer of Au was deposited by e-beam evaporation. After that, Au-coated samples were activated by plasma treatment (20 min, 30 W RF power, 1000 mTorr O_2_) and started the spin coating right after plasma treatment. Thenceforth, for coating hydrogels of different thicknesses, different spin-coating parameters were used, followed by cross-linking, as explained in Solution Preparation, Film Thickness Optimization, and Photopolymerization. The final gold layer (25–30 nm) was deposited on top of the hydrogel layer by a thermal evaporation system.

### Optical Measurements

Microscopic reflectance measurements were performed with a multifunctional WITec alpha300C confocal microscope. The samples with different hydrogel thicknesses were illuminated with a broadband light source (LDLS EQ-99X) through a Zeiss EC “Epiplan” DIC, 20× objective (NA = 0.4, WD = 3.0 mm). The reflected light was collected through the same objective and coupled to spectrometers via an optical fiber. For the spectral range of 400–900 nm, we used Ocean Optics Flame UV–vis spectrometer with 1.33 nm full width at half-maximum (FWHM) spectral resolution for detecting the spectral response from the fabricated sample. The samples were measured at room conditions (23 °C, 28% room RH - dry state) and after immersion in deionized water for 5, 10, and 20 min (wet state). Here, the relatively long hydrogel swelling time is due to the MIM structure, as the Au layers on the top/bottom of the hydrogel restrict water molecules from penetrating into the cross-linked polymer network.

Additionally, measurements in a controlled humid environment were performed using Linkam Scientific LTS420-H stage with RH95 humidity controller. Reflectance spectra were measured using the same WITec microscope and the samples were illuminated with a broadband light source. We utilized a 2.5× air objective with a relatively long working distance to enable the focusing of the optical field onto the samples embedded within the Linkam Scientific LTS420-H stage. For the PL measurement of the samples, we utilized a 532 nm laser to excite the samples to attain the emission peak intensity of the RhB dye incorporated within the MIM structure utilizing a 532 nm long-pass filter (LPF). The response of the samples was coupled to an optical fiber connected to an Ocean Optics Flame UV–vis spectrometer for PL measurement.
